# Corrective Osteotomy With Volar Plating of Distal Radius Malunions Using CT‐Based 3D Modeling: A Case Series of Four Patients

**DOI:** 10.1155/cro/4780901

**Published:** 2026-11-15

**Authors:** Carl Christian Holkgaard Burvil, Emil Toft Petersen, Janni Kjærgaard Thillemann, Jan Duedal Rölfing, Maiken Stilling

**Affiliations:** ^1^ AutoRSA Research Group Orthopaedic Research Unit Aarhus University Hospital Aarhus N Denmark auh.dk; ^2^ Department of Clinical Medicine Aarhus University Aarhus Denmark au.dk; ^3^ Department of Orthopedic Surgery Aarhus University Hospital Aarhus N Denmark auh.dk; ^4^ Department of Orthopedic Surgery Goedstrup Hospital Herning Denmark

**Keywords:** “fractures malunited”, “osteotomy”, “printing three-dimensional”, “radiostereometric analysis”, “wrist fractures”

## Abstract

Distal radius malunion is the most common complication following distal radius fractures and may lead to reduced forearm rotation, distal radioulnar joint (DRUJ) instability, and wrist pain. Corrective osteotomy is particularly challenging in complex three‐dimensional (3D) deformities. We evaluated the feasibility and outcomes of CT‐based 3D planning for corrective osteotomy in four patients with symptomatic distal radius malunion. Surgical planning was based on mirrored 3D models of the contralateral healthy radius. Two patients underwent simultaneous foveal triangular fibrocartilage complex (TFCC) reinsertion, while a third underwent delayed TFCC reinsertion combined with ulnar shortening osteotomy. Patients were assessed preoperatively and at 1‐year follow‐up using CT‐based 3D bone models, dynamic radiostereometric analysis (dRSA) of DRUJ kinematics, radiographic parameters, clinical examination, and patient‐reported outcome measures (QuickDASH and PRWHE). Anatomical correction improved in all patients, with the greatest improvements observed in rotational alignment. Radiographic parameters improved in all cases, although adequate radial length was restored in only two patients. QuickDASH scores improved in three patients, and PRWHE scores improved in all four. DRUJ kinematics demonstrated improved stability in two patients, no change in one, and worsening in one. Grip strength remained unchanged, while wrist range of motion improved in one patient. CT‐based 3D planning was feasible in all cases and facilitated accurate correction of complex distal radius malunions, particularly rotational deformities. Bony realignment improved DRUJ stability with and without TFCC reinsertion but did not restore normal kinematics in all patients. These findings suggest that TFCC stabilization should be reserved for patients with persistent symptomatic DRUJ instability after osteotomy healing and rehabilitation.

## 1. Introduction

Malunion is the most common complication of distal radius fractures (DRFs) [1, 2]. Nonoperative treatment of DRF may result in malunion in up to 24% [3], whereas operative treatment reduces the malunion rate to 10% [3–5].

A commonly reported symptom in patients with radius malunions is compromised forearm rotation [6]. In addition, a triangular fibrocartilage complex (TFCC) lesion is a frequent concomitant injury with radius fractures, which may lead to distal radioulnar joint (DRUJ) instability and pain [7–9]. Positive ulnar variance resulting from radial shortening may further contribute to persistent wrist pain [4, 10, 11]. As such, malunion may restrict hand and arm function during daily activities [6, 12].

The underlying bony deformities can be uni‐, bi‐, or multiplanar, and osteotomies must address all planes, that is, axis deviations, length discrepancies, and torsional malalignment [2].

Consequently, complete restoration of normal anatomy is challenging to achieve using freehand osteotomies and internal fixation. When the contralateral forearm is healthy, it can serve as a template for malunion correction [13]. Individualized 3D surgical planning and patient‐specific implants are becoming increasingly common for osteotomies, despite a lack of robust evidence supporting their superiority over freehand osteotomies and standard implants for fixation [14].

DRUJ stability depends on both bony congruency and ligamentous support from the TFCC, underscoring the critical importance of restoring normal bone anatomy and ligament integrity for normal joint function. DRUJ kinematics can be evaluated using dynamic radiostereometry (dRSA), a highly precise and validated imaging method that utilizes anatomical bone models generated from computed tomography (CT) [15–17]. Notably, the DRUJ kinematics before and after corrective osteotomy for radius malunion have not been studied previously.

The aim of this study was to evaluate outcomes before and after radius malunion correction using patient‐specific surgical planning by comparing radius bone anatomy using CT‐based 3D models, DRUJ kinematics, radiographic and clinical evaluations, and patient‐reported outcomes (PROMs).

## 2. Materials and Methods

In the period between October 2020 and February 2022, four patients were treated in our institution for malunion of distal radius or radial shaft fractures using patient‐specific surgical planning and correction. These were the first four patients with distal radius or radial shaft fracture malunions treated at our institution in the study period. Patient‐specific 3D planning was selected based on clinical assessment of the complexity with multiplanar malunion and the anticipated benefit of detailed preoperative planning. Patients presented with symptoms in terms of pain, reduced forearm rotation, and DRUJ instability (Table 1). Exclusion criteria were a previous fracture of the contralateral forearm (radius and ulna), concomitant ulnar deformities, and pregnancy. The surgical planning consisted of several steps (Figure 1), and patients were followed for a minimum of 12 months (range 12–37 months; Table 1). Patients consented to participation and the usage of their pseudonymized data for publication. The project was approved by the department. The patients were followed with standard pre‐ and postoperative examinations in our department as part of quality assurance. The Helsinki II declaration was followed.

**Table 1:** Patient demographics.

Patient	1	2	3	4
Age (years)	24	26	39	17
Sex	Female	Male	Male	Male
Injured side	Left	Left	Right	Left
Time from injury to corrective surgery (years)	10.3	3.7	3.5	2.8
Follow‐up time after radius corrective surgery (months)	14	12	37	24
Treatment indication	↓ supination, pain, DRUJ instability	↓ supination, pain, DRUJ instability	Pain, DRUJ instability	↓ supination, pain, DRUJ instability
Fracture type	Distal radius shaft fracture	Galeazzi fracture	Distal radius fracture	Distal radius shaft fracture
Other preoperative conditions	None	None	Primary open foveal TFCC reinsertion 2 years before referral for corrective radius osteotomy	Midcarpal instability
Arthroscopic TFCC injury type	Foveal	Foveal	Foveal	Arthroscopy not performed
TFCC repair	Primary open foveal TFCC reinsertion (suture anchor) concomitant with corrective radius osteotomy	Primary open foveal TFCC reinsertion (suture anchor) concomitant with corrective radius osteotomy	Secondary open foveal TFCC reinsertion (bone tunnel) and ulna shortening osteotomy 2 years after corrective radius osteotomy	None

**Figure 1:**
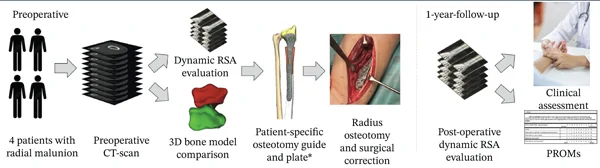
Overview of the preoperative planning, surgical procedure, and follow‐up. Preoperative CT scans were used for 3D reconstruction planning with mirroring to the contralateral radius to design patient‐specific osteotomy guides and plates. DRUJ kinematics were assessed with dynamic RSA pre‐ and postoperatively. One‐year follow‐up included clinical examination and patient‐reported outcomes. ∗In Patient 3, a standard Distal Radius VariAx2 plate was used.

### 2.1. Surgical Technique

After Henry′s surgical approach was employed to access the distal radius, the patient‐specific guide (IPS Gate, KLS Martin, Tuttlingen, Baden‐Württemberg, Germany) was positioned to achieve an optimal fit on the bone and subsequently fixed with K‐wires; all drill holes were then made according to the guide before completion of the osteotomy. The planned preoperative radius correction was incorporated into the correction guide; thus, when the patient‐specific titanium osteosynthesis plate (IPS Gate, KLS Martin, Tuttlingen, Baden‐Württemberg, Germany) was fixed to the radius with screws, the radius malunion was corrected (Figure 2).

**Figure 2:**
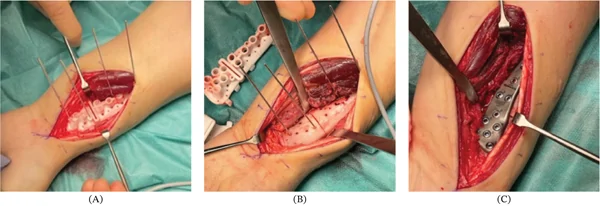
Surgical technique illustrated here in Patient 4. (A) 3D printed cutting and drill guide fixed to the radius with k‐wires. (B) Saw cut through the radius. (C) Patient‐specific plate mounted on the radius.

For Patient 3, a standard Distal Radius VariAx2 plate (Stryker, Portage, Michigan, United States) was used, as 3D planning revealed a uniplanar deformity, i.e., dorsal tilt (Table 2). An iliac crest graft was harvested and used to fill the osteotomy defect in two cases (Patients 1 and 2).

**Table 2:** Preoperative and final follow‐up radiographic outcomes measured on conventional radiographs.

Measure	Preoperative	Postoperative
Ulnar variance		
Patient 1	+1.5 mm	0 mm
Patient 2	+3.5 mm	+1.5 mm
Patient 3	+1.7 mm	−1 mm^a^
Patient 4	+1.0 mm	+2.4 mm
Radial inclination		
Patient 1	11°	27°
Patient 2	23°	23°
Patient 3	16°	20°
Patient 4	21°	25°
Radial tilt (+volar/−dorsal)		
Patient 1	−11°	5°
Patient 2	4°	9°
Patient 3	−18°	1°
Patient 4	13°	9°

^a^After secondary ulna shortening.

For Patients 1 and 2, arthroscopy was performed after the corrective radius osteotomy and confirmed the presence of a foveal TFCC lesion, which was in agreement with preoperative clinical findings of DRUJ instability as assessed with the Ballottement test. Therefore, an open foveal TFCC reinsertion was performed using a Mitek 2‐0 suture anchor (DePuy, Raynham, Massachusetts, United States) with confirmed intraoperative stabilization of the DRUJ. Postoperatively, both patients were immobilized in an above‐elbow cast for 6 weeks. Thereafter, the wrist was protected by a removable splint for an additional 4 weeks, while active unloaded pronation and supination exercises were initiated, as the patients followed the rehabilitation protocol for TFCC reinsertion at our institution.

For Patients 3 and 4, only slight intraoperative TFCC instability was present after corrective radius osteotomy, and wrist arthroscopy was not performed during the radius‐malunion corrective surgery. Two years following the radius osteotomy, Patient 3 underwent wrist arthroscopy and ulnar shortening osteotomy due to persistent ulnar‐sided pain coherent with ulnar impaction syndrome (positive ulna variance) and DRUJ instability. A foveal TFCC lesion was identified and treated by open foveal TFCC reinsertion using two bone tunnels and FiberWire 2‐0 mattress sutures (Arthrex, Munich, Germany). This patient followed the same immobilization and rehabilitation regimen as the previous cases. Patient 4 declined secondary arthroscopy and TFCC reinsertion, despite DRUJ instability on the Ballottement test and ulnar‐sided pain during load‐bearing activities, likely related to positive ulnar variance and a TFCC lesion.

### 2.2. CT

All patients had a CT scan of both forearms prior to surgery. At follow‐up, the CT scan was repeated for the injured side. Patient‐specific 3D surface and volume bone models of the radius and ulna were segmented from the CT scans. An anatomical coordinate system was applied to each bone model to estimate joint kinematics from the dRSA analysis [18]. The pre‐ and postoperative bone models of the injured arm were matched to the contralateral mirrored side proximal to the fracture site using custom‐made automated software (AutoRSA Research Group, Aarhus University Hospital, Denmark).

In collaboration with external engineers (IPS Gate, KLS Martin, Tuttlingen, Baden‐Württemberg, Germany), the radius osteotomy site and bone correction were determined (Figure 3), and a plastic drill‐and‐osteotomy guide and a patient‐specific 2.8 mm titanium osteosynthesis plate were designed and 3D printed (IPS Gate, KLS Martin, Tuttlingen, Baden‐Württemberg, Germany).

**Figure 3:**
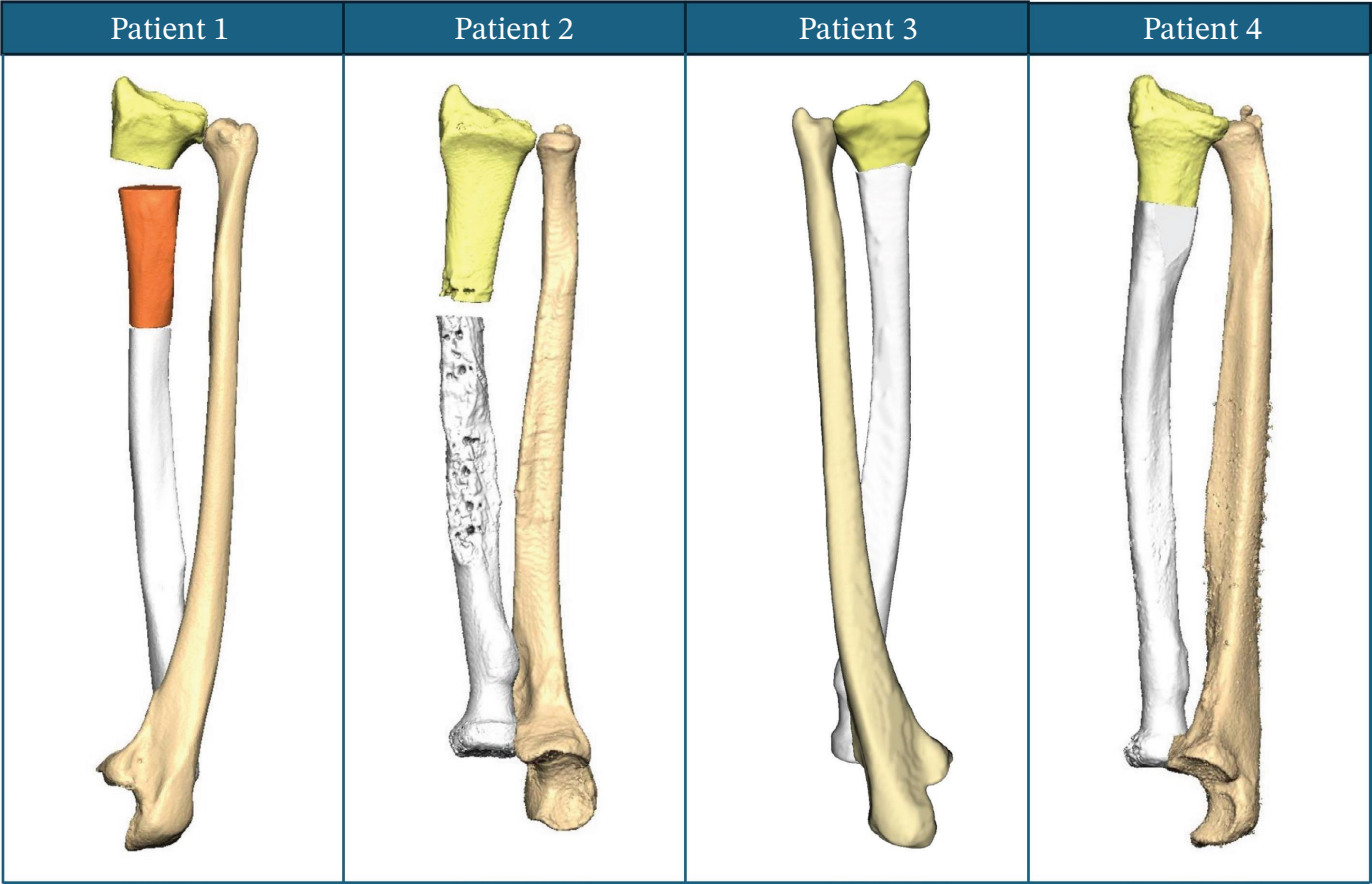
Correction plan with visualization of the osteotomy and the radial lengthening.

### 2.3. Dynamic Radiostereometry (dRSA)

For dRSA, two synchronized ceiling‐mounted x‐ray tubes captured pulsed images (15 frames per second) of the DRUJ during two pressure and motion tests (Figure 4). For details regarding the setup, we refer to previous literature [16]. The dRSA images were analyzed using an automated custom software system (AutoRSA, Aarhus University Hospital, Denmark) that references the 3D bone models for each image frame. The injured forearm was evaluated both preoperatively and 1 year postoperatively, while the contralateral forearm was assessed only preoperatively.

**Figure 4:**
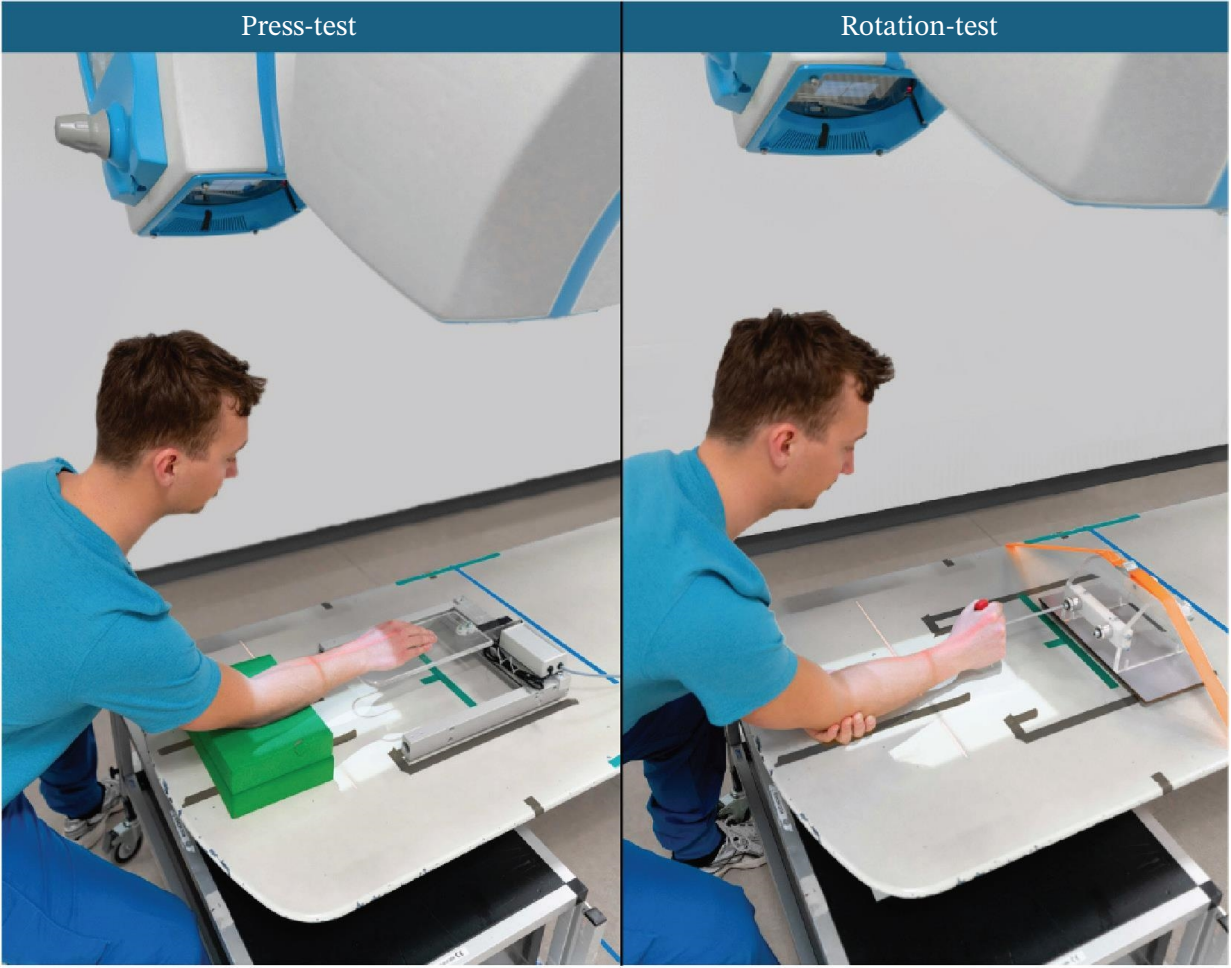
dRSA setup during a press test and a rotation test [16].

### 2.4. DRUJ Kinematics (Press Test)

During the dRSA recording, patients performed a press test as described by Thillemann et al. [16]. The patients were positioned in a standardized manner with elbow support on a foam pillow while applying hand pressure to a custom‐made unidirectional radiolucent platform (Figure 4). The patients were instructed to gradually apply force to the hypothenar region until maximum pressure was reached, then release the force, completing one press test motion cycle. In the current study, we used “offset” as a kinematic outcome, which indicates the position of the ulna head center on a line from the volar to the dorsal rim of the radial sigmoid notch [16].

### 2.5. DRUJ Kinematics (Unloaded Active Forearm Rotation Test; “Rotation Test”)

During the dRSA recording, the patients were positioned with the shoulder adducted, the elbow flexed, and supported by the contralateral hand (Figure 4). They were instructed to grab and turn a handle in a custom‐made device. The handle was without resistance (0 N torque). The patients then performed a gliding sequence of rotational movements: from neutral to supination, supination to neutral, neutral to pronation, and pronation back to neutral.

### 2.6. Radiographs

Posteroanterior and lateral radiographs were obtained for all patients both pre‐ and postoperatively (Figure 5). The ulnar variance was measured with Gelberman′s technique [20] as it is the most reliable [21]. Further, radial inclination [22] and volar/dorsal tilt were measured [23]. Normal values were considered to be ulnar variance 0.74 ± 1.46 mm [24], radial inclination 23° (range 13°–30°) [25], and volar tilt 11° (7°–15°) [26].

**Figure 5:**
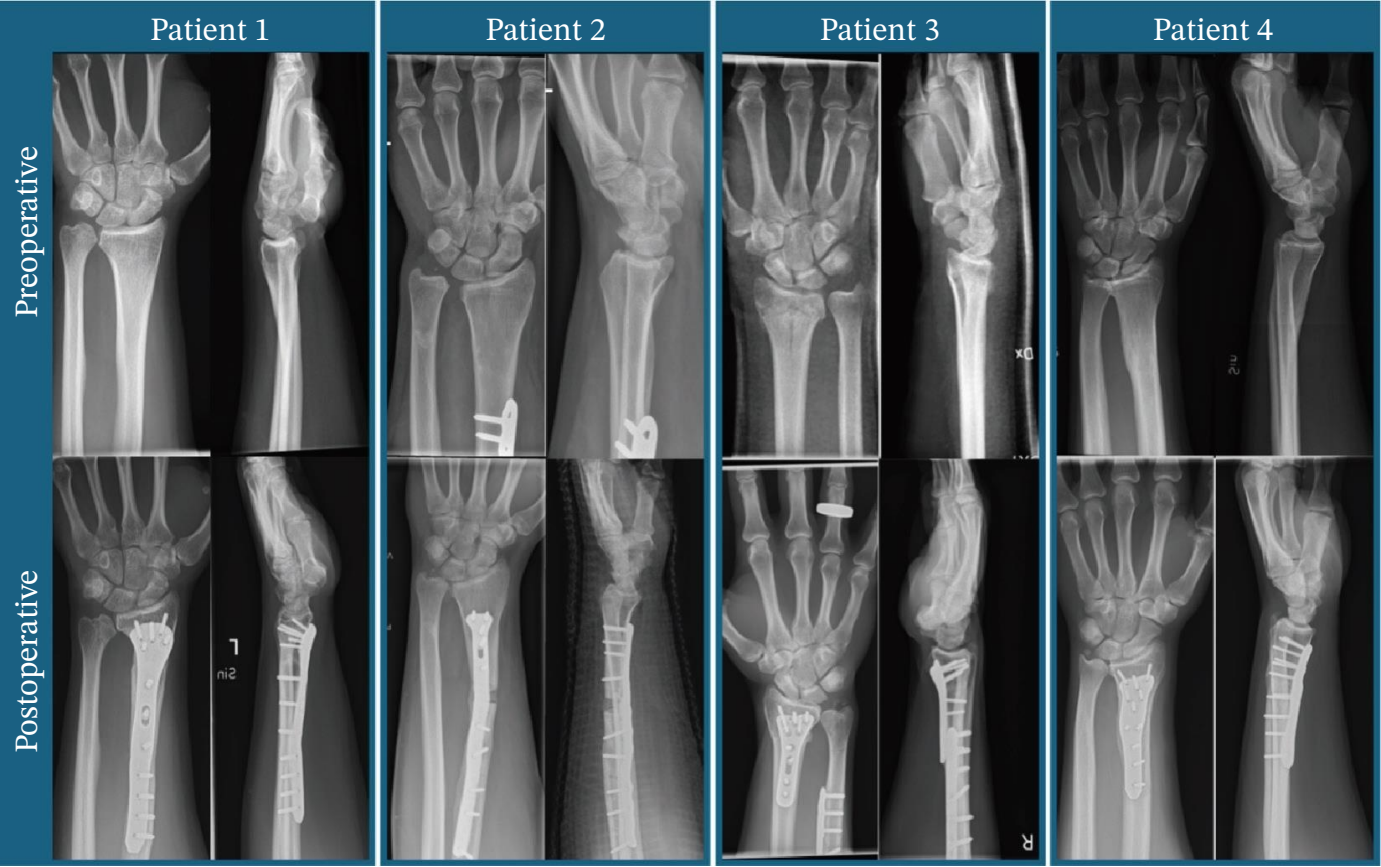
Preoperative and postoperative anterior‐posterior and lateral radiographs. Postoperative radiographs of Patients 1 and 4 were obtained 1 year after the radius osteotomy. Radiographs of Patient 2 were obtained 6 months after the radius osteotomy, whereas those of Patient 3 were obtained 6 months after ulnar shortening osteotomy and approximately 3 years after the radius osteotomy [19].

### 2.7. Clinical Examination

The surgeon performed the clinical evaluation. Grip strength was measured with a digital dynamometer, and rotation force was assessed using a forearm torque dynamometer (Baseline, Fabrication Enterprises Inc., White Plaines, New York, United States), with results reported as an average of three measurements. Range of motion (ROM) in the wrist and DRUJ of both the injured and contralateral forearm were measured using a goniometer.

### 2.8. Quick Disabilities of Arm, Shoulder, and Hand (QuickDASH) and Patient‐Rated Wrist and Hand Evaluation (PRWHE) Questionnaires

Before corrective surgery and at follow‐up, all patients were asked to complete the QuickDASH and the PRWHE questionnaires. The QuickDASH is an 11‐item shortened version of the 30‐item DASH, designed for self‐assessment of physical capabilities and symptoms in individuals with upper limb musculoskeletal issues. The overall QuickDASH score is calculated by averaging the responses, with scores ranging from 0 to 100, where higher scores indicate greater disability [27]. The minimally clinically important difference (MCID) for longitudinal changes for the QuickDASH score is 14 points for DRF [28]. The PRWHE questionnaire, comprising 15 items, enables individuals to assess their wrist pain and functional impairment on a scale from 0 (*best*) to 10 (*worst*). The overall PRWHE score, which combines pain (0–50) and functional (0–50) assessments, spans from 0 to 100, where a higher score reflects more pain and reduced hand/wrist functionality. The MCID for the PRWHE score is 11.5 points for DRFs [29]. At a minimum of 1 year postoperatively (last follow‐up), patients reported their subjective evaluation of the surgical outcome, including willingness to repeat the treatment, overall satisfaction, and pain compared to preoperative levels, using a 0–10 scale.

### 2.9. Complications

Intraoperative and postoperative complications, including malunion, neuropathies, infection, and secondary surgery, were registered.

## 3. Results

The mean age of the four patients at the time of surgery was 27 years (range 17–39). Patient demographics are presented in Table 1. The mean postoperative follow‐up after the corrective osteotomies was 22 months (range 12–37).

### 3.1. Radiographic Outcomes

All patients had positive ulnar variance at baseline. In three patients, the ulnar variance was decreased, whereas in Patient 4, it increased by 1.4 mm after 3D corrective surgery of the radius. In two patients, neutral or negative ulnar variance was achieved postoperatively; however, in the remaining two patients (Patients 2 and 4), a positive ulnar variance persisted on radiographs, despite the fact that 3D planning of the radius correction on CT scans is aimed at achieving a slightly negative ulnar variance. In Patient 3, the achievement of negative ulnar variance (−1 mm) was obtained after secondary surgery with ulna shortening osteotomy.

Radial inclination increased with 3D correction of the radius malunion and was within normal parameters for all patients at follow‐up. Radial tilt was with dorsal angulation in two patients, and volar angulation in two patients at baseline, but was corrected towards normal values in all patients (Table 2 and Figure 5).

### 3.2. 3D, Mirrored Bone Models

At baseline, all four patients had confirmation of radius malunion, as assessed by 3D bone mirroring of the contralateral side (Figure 5). All patients had improvement in all three axes towards the uninjured contralateral reference, but surprisingly not to a 100% perfect fit. The correction of rotation was very close to the planned correction (Figure 6).

**Figure 6:**
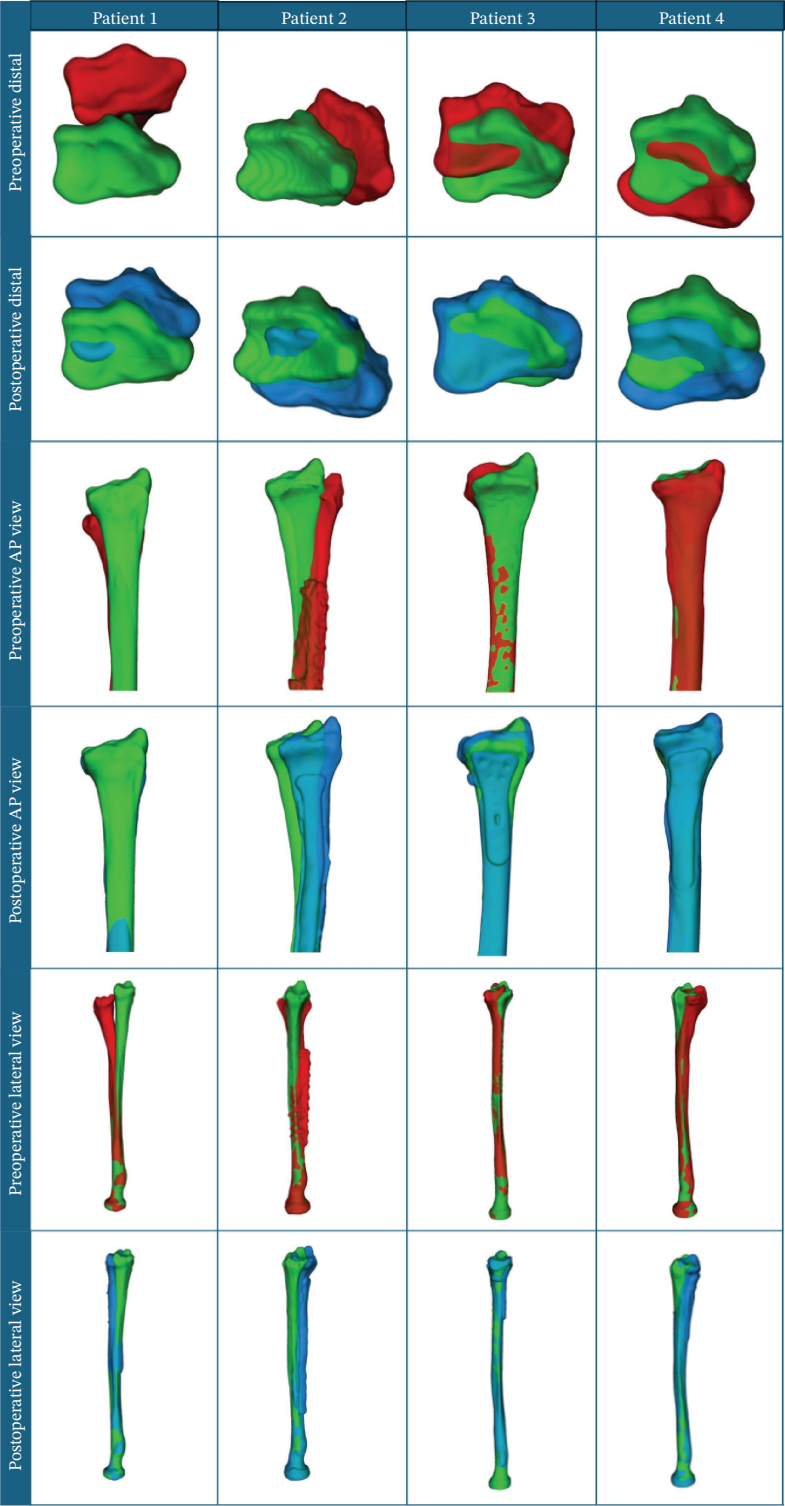
Correction of radius malunion displayed from a distal view, posterior‐anterior view, and a lateral view. CT surface models of the preoperative radius (red) and the postoperative radius (blue) mirrored with the uninjured contralateral radius (green).

### 3.3. Kinematic Results (dRSA)

The kinematic results for the press test and the rotation test are presented in Figures 7 and 8 and Table 3.

**Figure 7:**
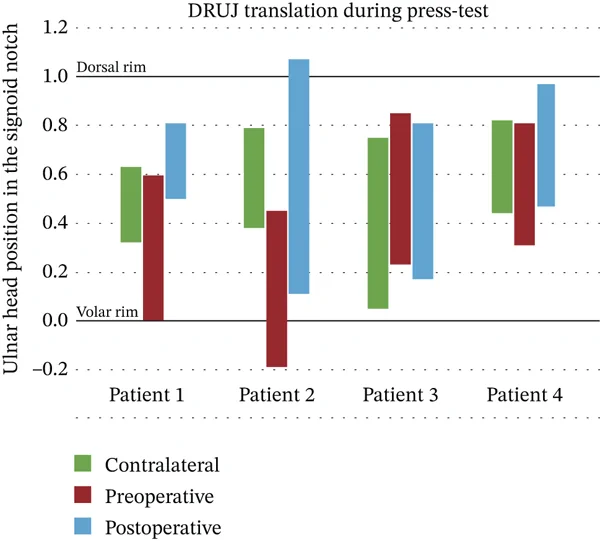
Press test displaying the range of translation as the position of the ulnar head center from the volar (0) to the dorsal rim (1) of the radial sigmoid notch. The preoperative DRUJ (red), the postoperative DRUJ (blue) after radius malunion correction, and the contralateral DRUJ (green) are displayed.

**Figure 8:**
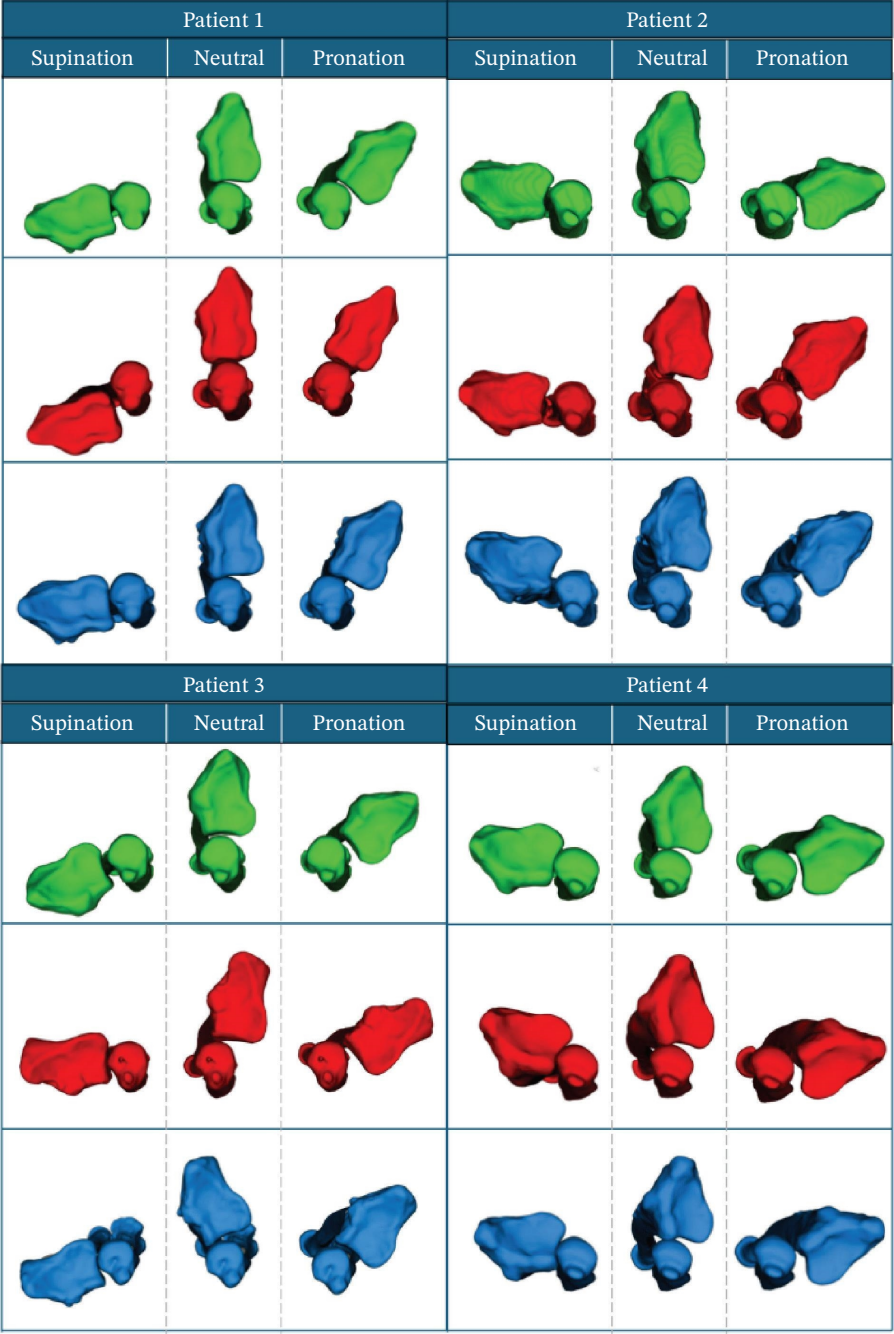
Rotation test with no load (0 N) showing the position of the ulnar head in the sigmoid notch of the DRUJ. CT surface models show the preoperative DRUJ (red), the postoperative DRUJ (blue) after radius malunion correction, and the contralateral DRUJ (green).

**Table 3:** Kinematic values of the DRUJ in the injured and contralateral forearm, before and after surgery, evaluated during a nonloaded (0 N) rotation test and press test.

	Preoperative	Postoperative	Contralateral healthy arm
Patient 1			
Supination (°)	66°	40°	60°
Pronation (°)	57°	62°	74°
Total ROM (°)	123°	102°	134°
Min offset^a^	0.00	0.50	0.32
Max offset	0.60	0.81	0.63
Total translation^b^	0.60	0.31	0.31
Patient 2			
Supination (°)	66°	65°	73°
Pronation (°)	55°	51°	83°
Total ROM (°)	121°	116°	156°
Min offset	−0.19	0.11	0.38
Max offset	0.45	1.07	0.79
Total translation	0.64	0.96	0.41
Patient 3			
Supination (°)	75°	67°	84°
Pronation (°)	62°	66°	79°
Total ROM (°)	137°	133°	163°
Min offset	0.23	0.17	0.05
Max offset	0.85	0.81	0.75
Total translation	0.62	0.64	0.70
Patient 4			
Supination (°)	52°	78°	91°
Pronation (°)	76°	62°	53°
Total ROM (°)	128°	140°	144°
Min offset	0.31	0.47	0.44
Max offset	0.81	0.97	0.82
Total translation	0.50	0.50	0.38

^a^Offset was defined as the position of the ulna head center projected orthogonally on a line from the volar (0) to the dorsal rim (1) of the radial sigmoid notch.

^b^Total translation: translation of the ulnar head center on the radial sigmoid notch line during a press test.

#### 3.3.1. Patient 1

The press test showed preoperatively that the ulnar head translated into the volar portion of the sigmoid notch. Radius malunion correction and primary TFCC reinsertion increased DRUJ stability, but shifted the position of the ulnar head to a dorsal position (Figure 7 and Table 3).

The rotation test showed a preoperative tendency of volar subluxation of the ulnar head in the sigmoid notch in supination, which was corrected postoperatively. The postoperative ulnar head position in pronation was similar to the contralateral DRUJ. Radius malunion correction resulted in a loss of 26° supination and a gain of 5° pronation (Figure 8 and Table 3).

#### 3.3.2. Patient 2

The press test showed preoperatively that the ulnar head translated into the volar portion of the sigmoid notch, with subluxation over the volar sigmoid notch rim at maximum applied pressure. Clinically, the ulnar head appeared fixed in a volar position. The radius malunion was slightly overcorrected with a slight volar shaft angulation, resulting in a shift of the position and translation of the ulnar head into the sigmoid notch (Figure 7 and Table 3). However, the translation path was wide, indicating a lack of DRUJ stabilization despite foveal TFCC reinsertion.

The rotation test showed the ulnar head in a dorsal position of the sigmoid notch in supination and neutral DRUJ rotation, both preoperatively and after radius malunion correction. The postoperative ulnar head position in pronation was also more dorsal than in the contralateral DRUJ. Throughout the rotation test, there was a gap between the ulna head and the sigmoid notch, which did not improve postoperatively (Figure 8). Radius malunion correction resulted in a loss of 1° supination and a loss of 4° pronation (Table 3).

#### 3.3.3. Patient 3

The press test showed translation of the ulnar head in the central part of the sigmoid notch, which was similar pre‐ and postoperative to radius malunion correction and secondary ulna shortening with foveal TFCC reinsertion, and in comparison with the contralateral DRUJ (Figure 7 and Table 3).

The rotation test showed improvement of the ulnar head position in the sigmoid notch in neutral position and pronation after radius malunion correction (Figure 8).

Radius malunion correction resulted in a loss of 8° supination and a gain of 5° pronation (Table 3).

#### 3.3.4. Patient 4

The press test showed a preoperative central position and a narrow translation path of the ulnar head centrally in the sigmoid notch, which shifted to a more dorsal part of the sigmoid notch after radius malunion correction (Figure 7 and Table 3). Although clinically unstable in the DRUJ and midcarpal joint, the patient refrained from diagnostic wrist arthroscopy and further surgery.

The rotation test showed an unchanged position of the ulnar head in the sigmoid notch throughout forearm rotation (Figure 8).

Radius malunion correction resulted in a gain of 26° supination and a loss of 14° pronation (Table 3).

### 3.4. Clinical Examination

At follow‐up, grip strength was comparable to the contralateral side in two patients, while the remaining two patients achieved 49% and 63% of their contralateral grip strength, respectively (Table 4). Compared to preoperative values, Patient 1 experienced a 25% reduction in grip strength, Patient 2 showed a slight improvement, and Patient 3 had unchanged grip strength. Wrist flexion, extension, radial deviation, ulnar deviation, and pronation remained unchanged at the group level, but with individual differences (Table 5). Supination improved clinically relevant in two patients, was unchanged in one patient, and decreased 10° in one patient.

**Table 4:** Grip strength and torque force with corresponding registration of pain during grip strength measurement.

	Preoperative		Postoperative		Contralateral healthy arm	
	kg	NRS	kg	NRS	kg	NRS
Grip strength (kg)/pain (NRS)						
Patient 1	29.2 kg	4	16.3 kg	N/A	33.1 kg	0
Patient 2	22.2 kg	5	25.1 kg	6	55.3 kg	0
Patient 3	55.4 kg	0	54.3 kg	2	51.2 kg	0
Patient 4	N/A	N/A	63.9 kg	6	57.2 kg	5
Torque force pronation (kg)/pain (NRS)						
Patient 1	99 kg	2	N/A	N/A	87.3 kg	0
Patient 2	113 kg	9	130 kg	7	290 kg	1
Patient 3	124 kg	2	165 kg	4	159 kg	0
Patient 4	N/A	N/A	162 kg	9	134 kg	0
Torque force supination (kg)/pain (NRS)						
Patient 1	62 kg	3	N/A	N/A	115 kg	0
Patient 2	107 kg	8	156 kg	5	240 kg	1
Patient 3	127 kg	2	177 kg	3	150 kg	0
Patient 4	N/A	N/A	289 kg	6	299 kg	1

Abbreviation: NRS: numeric rating scale 0–10.

**Table 5:** Radiocarpal and DRUJ ROM measured with a goniometer pre‐ and at the final postoperative follow‐up.

Measure	Preoperative	Postoperative	Contralateral healthy arm
Flexion			
Patient 1	55°	60°	80°
Patient 2	30°	50°	55°
Patient 3	45°	65°	75°
Patient 4	90°	70°	90°
Mean	55°	61.3°	75°
Extension			
Patient 1	80°	75°	80°
Patient 2	30°	65°	75°
Patient 3	70°	85°	80°
Patient 4	90°	75°	90°
Mean	67.5°	75.0°	81.3°
Ulnar deviation			
Patient 1	25°	35°	35°
Patient 2	20°	25°	40°
Patient 3	30°	30°	40°
Patient 4	35°	35°	35°
Mean	27.5°	31.3°	37.5°
Radial deviation			
Patient 1	20°	20°	15°
Patient 2	10°	15°	35°
Patient 3	20°	25°	20°
Patient 4	25°	25°	25°
Mean	18.8°	21.3°	23.8°
Pronation			
Patient 1	90°	90°	90°
Patient 2	85°	90°	85°
Patient 3	85°	75°	90°
Patient 4	90°	75°	90°
Mean	87.5°	82.5°	88.8°
Supination			
Patient 1	90°	80°	90°
Patient 2	50°	80°	70°
Patient 3	90°	90°	90°
Patient 4	65°	90°	90°
Mean	73.8°	85.0°	85°

### 3.5. PROMs

At follow‐up, the QuickDASH score improved in three of the four patients with a mean improvement of 5.5 points (range −5 to 16). The PRWHE improved in all patients by a mean of 13.5 points (range 3–20), with clinically relevant improvement in Patients 1 and 2 (Table 6).

**Table 6:** Patient‐reported outcomes (PROM) pre‐ and at the final postoperative follow‐up.

	**Preoperative**			**Postoperative**		
QuickDASH score						
Patient 1	43			34		
Patient 2	45			50		
Patient 3^a^	36			20		
Patient 4	30			27		
Mean	38.5			33		
PRWHE score	**Pain**	**Function**	**Total**	**Pain**	**Function**	**Total**
Patient 1	39	17	56	28	8	36
Patient 2	38	33	71	28	21	49
Patient 3^a^	22	15	37	17	11	28
Patient 4	21	10	31	19	9	28
Mean	30	19	49	23	12	35
Patient subjective evaluation	**Willingness to repeat surgery**		**Satisfaction with the result**		**Pain compared to before the surgery**	
Patient 1	Yes		Very satisfied		Reduced	
Patient 2	Yes		Very satisfied		Reduced	
Patient 3^a^	Yes		Neither nor		Unchanged	
Patient 4	No		Neither nor		Increased	

Abbreviations: PRWHE, Patient‐Rated Wrist and Hand Evaluation score; QuickDASH, Quick Disabilities of Arm, Shoulder, and Hand score.

^a^The final postoperative results for Patient 3 after secondary ulnar shortening and TFCC‐reinsertion.

Patients 1 and 2 reported willingness to repeat the surgery, high satisfaction, and the overall pain score was reduced compared to before surgery. Patient 3 reported willingness to repeat surgery but also reported unaltered pain and thus an overall neutral satisfaction regarding surgery. Patient 4 was not willing to repeat surgery but reported neutral satisfaction.

### 3.6. Complications

There were no nonunions or infections. Patient 1 had temporary median nerve neuropathy (sensory neuropraxia) for 2–3 months. Patient 3 underwent secondary ulna shortening and TFCC reinsertion 12 months after radius malunion correction.

## 4. Discussion

This case study presents the results of anatomical correction of distal radius malunion using CT‐based 3D mirroring of the healthy contralateral arm. Generally, all malunions improved in axial angulation, but it was especially beneficial for correcting rotational malalignment.

### 4.1. Anatomical Level of Radius Malunion

The anatomical level and severity of the radius malunion influence the planning strategy for triplane correction. For distal radius malunion, the indication for corrective osteotomy is the presence of persistent pain and functional impairment in combination with radiographic deformity [30]. Dorsal angulation and radial shortening leading to painful positive ulnar variance are described as the main factors limiting forearm rotation [6].

The deformity of distal radius malunion may be successfully assessed by radiographs and corrected with freehand osteotomy and standard volar locking plates [14], as in Case 3. Thus, patient‐specific 3D planning and custom‐made implants may not be necessary for every malunion, particularly in cases with predominantly uniplanar deformity and length discrepancy that can be adequately characterized and corrected using conventional 2D imaging and techniques. On the contrary, the deformity of more proximal radius malunions is difficult to assess on radiographs (2D) and necessitates CT scans and 3D correction planning for a more predictable outcome, as in Cases 1, 2, and 4. The indications for corrective osteotomy in more proximal radius malunion include angular deformity above 15°, or malrotation above 20°–30°, or radial shortening leading to restricted forearm pronation and/or supination, or painful DRUJ instability [31].

### 4.2. Radial Length and Ulnar Variance

Ulnar variance is a radiographic measure on conventional posterior‐anterior radiographs, which describes the relation between radius and ulna length. The agreement between 2D measurement methods and 3D‐based assessment of ulnar variance is not clear [32–34]. Cases 1 and 2 were planned with a triplane rotation correction, and despite a 10 mm radial lengthening in both cases (Figure 8), aiming to achieve a small negative ulnar variance, the final radiological results showed 0–1.5 mm positive ulnar variance. Changes in volar tilt or radial inclination during 3D planning may necessitate overcorrection of radial length, and/or simultaneous ulnar shortening, to ensure a slightly negative ulnar variance and avoid ulnar abutment [35]. Another option using patient‐specific plates may be to plan with intraoperative length adjustability, via two oval dynamic compression holes, to ensure appropriate ulnar variance.

### 4.3. Osteotomy and Bone Graft

Radial lengthening of up to 1 cm without bone graft, leaving a residual osteotomy gap, has been shown to heal well in young, healthy individuals with good bone quality [36, 37]. However, it is unknown whether any minor collapse of the osteotomy gap occurs during the healing period. In Patient 1 and 2, who were healthy and in their twenties, the planned radial bone length was not achieved despite the use of a 10 mm iliac crest bone graft to support the osteotomy gap (Figure 3). A double‐radius osteotomy resulting in a mobile bone wedge may provide better radius deformity correction, such as in Patient 1. However, the complexity of performing such a procedure freehand increases the risk of complications and supports 3D planning and correction. Furthermore, excessive radial lengthening carries a risk of neurovascular compromise due to traction, which may lead to neuropraxia, as in Case 1. Therefore, excessive radial lengthening is not recommended [38]. As such, a compromise for safe and sufficient correction of severely shortened radius malunions may include ulnar shortening [39]. However, routine two‐bone shortening is mainly indicated when a concomitant ulnar deformity requires correction.

### 4.4. Cutting Guide

Accomplishment of the planned osteotomy correction depends on precise guide placement for the determination of the osteotomy site. The surgeon relies blindly on the manufacturer′s correct production of CT‐based guides, in terms of size and shape, to perfectly fit the patient′s bone surface intraoperatively. Furthermore, the guide material must be sufficiently rigid to prevent unintended deformation, as any bending or torsion may increase the angulation or rotation of the predrilled holes and compromise the accuracy of the osteotomy and thus, the final correction. A double osteotomy may increase the likelihood of achieving precise anatomical bone correction when the deformity is prominent, located more proximally in the radius and accompanied by complex multiplanar malalignment. In cases involving closing‐wedge osteotomies, a guide design with two separate saw slots for the wedge osteotomy is more feasible, as it may provide a more reliable bone contact in the docking site. However, with a single closing‐wedge osteotomy, a simultaneous ulna shortening osteotomy may be required, increasing the complexity of the surgery.

### 4.5. DRUJ Stability and Kinematics

TFCC injuries are common in association with DRFs [9]. However, TFCC repair and reconstruction are contraindicated in the presence of residual radial deformity, as persistent bony incongruency may compromise DRUJ stability and lead to insufficiency of the soft‐tissue stabilizers, including the TFCC and interosseous membrane [40]. Even after anatomical correction of radial malunion and restoration of DRUJ congruency, instability may persist despite the TFCC seeming uninjured in MRI or arthroscopy. This finding is likely due to chronic elongation and insufficiency of these structures, secondary to long‐standing bony incongruency [40], and may explain the persistent DRUJ instability in Patient 3. In Patients 1 and 2, a standard open foveal TFCC reinsertion technique, as employed concomitantly with a one‐stage radius osteotomy, did not provide sufficient DRUJ stability at follow‐up. Even in Patient 3, who received a secondary foveal TFCC reinsertion, the DRUJ was not sufficiently stabilized. The optimal timing of TFCC repair or reconstruction—whether performed primarily (at the time of osteotomy) or secondarily—remains an unanswered question. In our practice, we now employ TFCC repair or reconstruction as a secondary procedure with a minimum 6‐month window after the malunion correction, which allows for bone healing and settlement of the DRUJ soft tissue stabilizers. A persistent gap in the DRUJ, as seen in Patient 2 despite primary TFCC reinsertion and in Patient 4, who refrained from TFCC reinsertion, indicates gross TFCC instability. For chronic TFCC lesions, TFCC reconstruction with a tendon graft is recommended [40, 41].

### 4.6. PROMs

Several studies investigating 3D surgical planning for distal radius malunion correction using a commercial KLS correction system, which we also have used in this study, have reported marked improvements in PRWHE scores [14, 42, 43]. We observed a mean improvement of 13.5 points, which is markedly lower than the 41, 40, and 51 points reported by Bodmer et al. [14], Schindele et al. [42], and Raad et al. [43], respectively. This discrepancy may partly be explained by the complexity and heterogeneity of our cohort, which consisted of four patients with severe preoperative malunions and concomitant persistent DRUJ instability, potentially limiting their postoperative improvement. Another likely contributing factor is the pronounced age difference between the cohorts: Our patients had a mean age of 27 years, whereas the comparative studies reported mean ages of 51, 56, and 44 years, respectively. Younger patients are known to have higher functional demands and postoperative expectations, which may adversely affect PROMs [44, 45], and might explain why the MCID was not met for QuickDASH. Importantly, in this case series, we observed no coherence between the degree of anatomical correction and PROMs or patient satisfaction. As such, other factors—such as concomitant TFCC injury or midcarpal instability—may have influenced the clinical outcome. This hypothesis warrants further verification in larger cohort studies.

### 4.7. Limitations

This study has several important limitations. First, the heterogeneity of the four cases limits the ability to draw generalized conclusions. Furthermore, these represent the first patients at our institution treated with 3D‐planned corrective osteotomy for distal radius malunion, introducing potential learning‐curve effects. Further, the concomitant TFCC reinsertion in two patients represents a potential confounding factor when interpreting postoperative DRUJ kinematics, as the contribution of the corrective radius osteotomy on DRUJ stability cannot be isolated.

Another limitation relates to the postoperative CT evaluation. The discrepancy between planned and achieved correction was greater than anticipated. We hypothesize that biological remodeling during the healing phase may contribute to this difference and that the interval between surgery and follow‐up CT imaging may influence the accuracy of bone model comparison. This is a subject warranting further investigation, as it might affect preoperative planning.

Despite using patient‐specific 3D planning and correction guides, designed by engineers in a commercial company, a match to the contralateral healthy arm′s anatomy was not achieved with 100% precision (Figure 6). It is unknown whether the reason is the template and plate design, inaccuracy during surgery, remodeling after surgery, or the use of the contralateral healthy radius as a template for correcting the injured radius. Studies assessing bilateral radial symmetry report a high degree of anatomical concordance between the contralateral radii [13], whereas others have demonstrated side‐to‐side differences and have questioned the suitability of the healthy arm as an ideal template [46].

Several studies have demonstrated excellent functional outcomes with standard volar locking plates [47], and although 3D‐assisted techniques may facilitate superior anatomical correction, this does not necessarily translate into improved PROMs. Similarly, improved anatomical alignment did not consistently translate into improved grip strength or ROM, which may reflect the complex nature of these cases, involving both bone deformity and associated soft tissue injuries.

Customized implants are also rather costly, and the cost‐effectiveness of patient‐specific 3D planning with custom‐made correction guides and osteosynthesis plates needs further investigation.

## 5. Conclusion

Three‐dimensional planning using the contralateral healthy radius as a template was feasible for corrective osteotomy of all radius malunions and was especially beneficial for correction of rotational malalignment. Bony realignment improved DRUJ stability with and without TFCC reinsertion, but it did not normalize to the healthy side in all patients. As such, we suggest that TFCC stabilizing procedures are reserved for cases of persistent; symptomatic DRUJ instability after osteotomy healing and rehabilitation is completed.

## Author Contributions

Carl Christian Holkgaard Burvil: writing—original draft, conceptualization, methodology, validation, data curation, investigation, and formal analysis. Emil Toft Petersen: software, methodology, formal analysis, investigation, data curation, supervision, and writing—review and editing. Janni Kjærgaard Thillemann: conceptualization, resources, and writing—review and editing. Jan Duedal Rölfing: resources and writing—review and editing. Maiken Stilling: conceptualization, methodology, supervision, investigation, resources, project administration, and writing—review and editing.

## Funding

No funding was received for this manuscript.

## Conflicts of Interest

The authors declare no conflicts of interest.
